# Programmed cell death 1 ligand (PD-L1) on T cells generates Treg suppression from memory

**DOI:** 10.1371/journal.pbio.3001272

**Published:** 2021-05-19

**Authors:** Alexandra Kazanova, Christopher E. Rudd

**Affiliations:** 1 Division of Immunology-Oncology, Centre de Recherche Hopital Maisonneuve-Rosemont (CR-HMR) Montreal, Canada; 2 Department of Microbiology, Infection and Immunology, Faculty of Medicine, Universite de Montreal, Montreal, Canada; 3 Department of Medicine, McGill University Health Center, Montreal, Canada

## Abstract

A recent study shows that programmed cell death 1 ligand (PD-L1) on activated T cells promotes their conversion to suppressive inducible regulatory T cells (iTregs), preferentially from among memory T cells. This new mechanism may normally protect against autoimmunity but is dysregulated in rheumatoid arthritis.

Immune checkpoint blockade (ICB) therapy involves the use of antibodies that block the interaction between inhibitory receptors, like programmed cell death 1 (PD-1) and its ligand, programmed cell death 1 ligand (PD-L1) (also known as B7-H1 or CD274). ICB with anti-PD-1 (Nivolumab and Pembrolizumab) and anti-PD-L1 (Atezolizumab) has revolutionized the treatment of cancer [1]. PD-1 is expressed on activated T cells where it generates negative signals that inhibit function and contribute to T cell exhaustion [2]. On the other hand, PD-L1/2 is broadly expressed on hematopoietic, parenchymal, and stromal cells including antigen-presenting cells (APCs) [2]. PD-L1 expression on APCs suppresses T cell immunity by engaging PD-1 in a process that contributes to the induction of regulatory T cells (Tregs) [3,4]. Tregs, defined by the expression of the transcription factor forkhead P3 (FOXP3), the high-affinity interleukin-2 receptor α (CD25), and the inhibitory receptor CTLA-4, protect against excess inflammation and autoimmunity [5]. There are 2 types of Tregs, one that is thymus derived, also known as natural occurring Tregs (tTregs), and another that is inducible in the peripheral T cell compartment and can be induced in vitro (iTregs) [6].

PD-L1 is also expressed on activated T cells where its function has been unclear. A study had previously demonstrated that PD-L1 ligation on CD4 T cells can prevent their activation and polarization [7]. A new study published in *PLOS Biology* by Fanelli and colleagues now shows that PD-L1 engagement on T cells actually promotes their conversion to iTregs [8]. Further, in a surprising twist, the authors noted that this conversion occurred primarily among the memory human T cell as defined by the phenotype CD4^+^CD25^−^CD45RA^−^CD45RO^+^. CD25^hi^FOXP3^hi^ Tregs expanded preferentially from the memory pool. Memory T cells are key to the immune system since they remain for prolonged periods after infections end and can be converted into effector T cells upon reexposure to antigen. PD-L1 conversion of this memory pool to Tregs would, therefore, act to limit recall responses by both inducing suppressor cells and reducing the size of the memory pool. This could have important consequences for the ability of the immune system to respond to infections and affect the efficacy of immune checkpoint immunotherapy against cancer.

The authors showed that antibody ligation of PD-L1 induced the expression of FOXP3, CD25, and CTLA-4 and that iTregs could be generated from PD-1 knockout CD4 T cells. Interestingly, cytometry by time-of-flight (CyTOF) analysis revealed that PD-L1 engagement increased the expression of other surface receptors such as CD69, CD28, OX40, and intracellular Ki67. More specifically, PD-L1 ligation generated a subset with low expression of Helios and high levels of inducible costimulator (ICOS), CD28, CCR4, and CTLA-4. In this context, CD28^hi^, ICOS^hi^, CTLA-4^hi^, and Helios have been described to define memory Tregs. In terms of intracellular signaling, the pathway involved the AKT–mTOR and MAP kinase pathways consistent with previous studies showing that CTLA-4 binds to phosphatidyl inositol 3-kinase (PI3K) which in turn activates AKT–mTOR [9], and where the truncation of PI3K signaling can induce Tregs [10]. Conversely, there was an increase in STAT3 and STAT5 phosphorylation, the latter known to participate in Treg development and function. Memory T cells produced high levels of the cytokine interferon gamma (IFN-γ) after crosslinking with antibodies to CD3 and PD-L1 which was hypothesized to contribute to the conversion of naïve T cells to iTregs by up-regulating PD-L1 (Fig 1).

**Fig 1:**
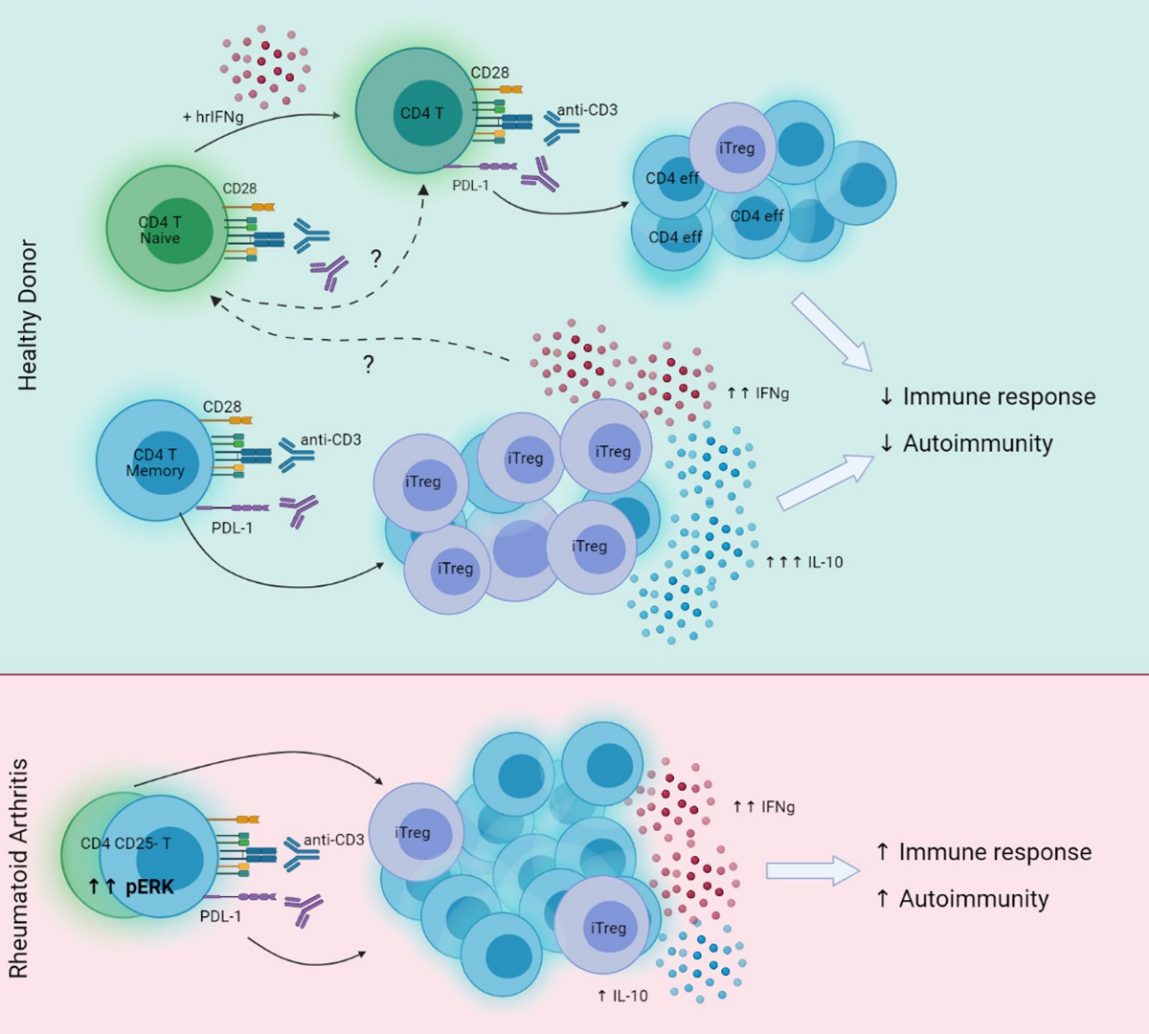
PD-L1 induction of iTregs from human memory CD4 T cells. Healthy donors’ (upper light green level) naive CD4 CD25− T cells (green cells) and memory CD45RO+CD45RA− CD4 cells (blue cells) were activated with plate-bound anti-CD3 and anti-PD-L1. Memory CD4 cells (blue cells) were the main source of highly suppressive iTreg (violet cells) with high production of IL-10. Same activation of CD4+CD25− memory and naive T cells from rheumatoid arthritis patients (lower pink level) failed to generate major suppressors of autoimmunity—Tregs. Created with biorender.com. hrIFNg, human recombinant interferon gamma; IFNg, interferon gamma; IL-10, interleukin 10; iTregs, inducible regulatory T cells; PD-L1, programmed cell death 1 ligand; pERK, phosphorylated ERK.

Lastly, with supportive clinical data, the authors showed that T cells from patients with rheumatoid arthritis (RA) were refractory to the acquisition of the iTreg phenotype. Fanelli and colleagues proposed that this resistance was mediated via high basal levels of phosphorylated ERK. In T cells from RA patients, PD-L1 crosslinking failed to reduce signaling via the ERK and AKT/mTOR/S6 pathways. Further, while PD-L1/CD3 crosslinking in healthy donors reduced pSTAT3 compared to CD28/CD3, the decrease in RA patients was more modest. Overall, these results suggested that PD-L1 signaling on memory T cells could play an important role in resolving inflammatory responses, maintaining a tolerogenic environment and its failure could contribute to ongoing autoimmunity.

The exciting work of Fanelli and colleagues also opens up other important questions. The full implications of recruitment from memory T cell, their more detailed identity as memory Tregs, their possible plasticity, and reconversion back to effector or memory T cells are unclear and remain to be explored. Future studies will benefit from a more refined definition of the nature of the memory T cells. Subsets include central memory (CM), effector memory (EM), and tissue-resident memory T cells (T_RM_) which can be defined with markers such as CD45RA, CD45RO, CD62L, CCR7, CD127, CD69, and the integrin CD103. As mentioned, the conversion of memory cells to Tregs could reduce the presence of memory T cells needed to mount a subsequent recall response to antigen. This could be of great importance to checkpoint blockade therapy where the binding of antibodies to PD-L1 (Atezolizumab) can promote antitumor immunity. However, in this scenario, the induction of suppressor cells and the reduction of memory T cells would be expected to reduce the benefits of this cancer therapy. The final outcome may depend on the relative efficiency of antibody engagement versus its ability to block the binding of natural ligand PD-1 to PD-L1. In this context, as a limitation to the study, antibodies alone were used to engage PD-L1, and so the relative efficacy of PD-1, or another PD-L1 binding receptor, CD80, versus antibody on the induction of iTregs needs to be resolved. It also remains to be determined which other mechanisms contribute to the suppressor function of iTregs besides the production of interleukin 10 (IL-10). One possibility is that the PD-L1–PD-1 pathway offers a mechanism by which T cells can regulate other T cells, an interaction that has previously been associated with reduced function. Further, it will be interesting to know whether PD-L2 has the same effect since this binding partner has generally been found less effective in engaging PD-1 negative regulation of T cell function. The main benefit of this intriguing new pathway may be in the control of autoimmune disorders where inflammatory responses would be reduced by the induction of iTregs.

## Abbreviations

APCs: antigen-presenting cells
CM: central memory
CyTOF: cytometry by time-of-flight
EM: effector memory
FOXP3: forkhead P3
ICB: immune checkpoint blockade
ICOS: inducible costimulatory
IFN-γ: interferon gamma
IL-10: interleukin 10
iTregs: inducible regulatory T cells
PD-1: programmed cell death 1
PD-L1: programmed cell death 1 ligand
PI3K: phosphatidyl inositol 3-kinase
RA: rheumatoid arthritis
Tregs: regulatory T cells
T_RM_: tissue-resident memory T cells
